# P-1894. Evaluation of an Outpatient Parenteral Antimicrobial Therapy (OPAT) Program Performance Against Published Key Performance Indicators (KPIs)

**DOI:** 10.1093/ofid/ofae631.2055

**Published:** 2025-01-29

**Authors:** Nicole Slain, Ryan P Mynatt, Ashley Logan, Donna R Burgess, Alisha Clemons, Armaghan-E Rehman Mansoor, Kathryn Ruf, Evelyn Villacorta Cari

**Affiliations:** University of Kentucky HealthCare, Lexington, Kentucky; University of Kentucky, Lexington, KY; University of Kentucky HealthCare, Lexington, Kentucky; UK HealthCare, Lexington, KY; University of Kentucky, Lexington, KY; University of Kentucky, Lexington, KY; University of Kentucky HealthCare, Lexington, Kentucky; University of Kentucky, Lexington, KY

## Abstract

**Background:**

Current OPAT guidelines emphasize identifying, reporting and improving key antimicrobial use and care coordination decisions. There is no clear guidance on benchmarking or consensus reporting recommendations. We examined our OPAT program’s performance against data published from Great Britain’s National Outcomes Registry System (NORS) as well as a meta-analysis on catheter related thrombosis (CRT) in OPAT (Figure 1).

**Figure:**
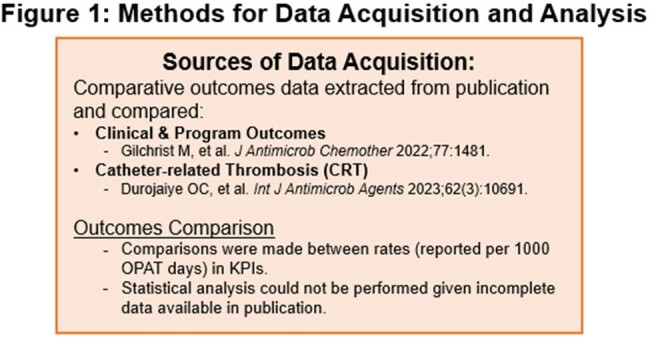

**Methods:**

Adult patients receiving OPAT from infectious diseases (ID) ambulatory and inpatient consult teams from June 2021 to July 2022 were included. Patient demographics, admission data, and OPAT outcomes were recorded as part of routine pharmacist workflow. Data were compared against those reported via the NORS registry as well as a meta-analysis evaluating CRT. Specific comparisons relative to reported rates and ranges were made in key clinical areas including infection type, clinical outcomes, OPAT completion, VAD events, adverse drug events (ADEs) and medication-related events. Given limitations to available published data, a statistical comparison was not performed.

**Figure:**
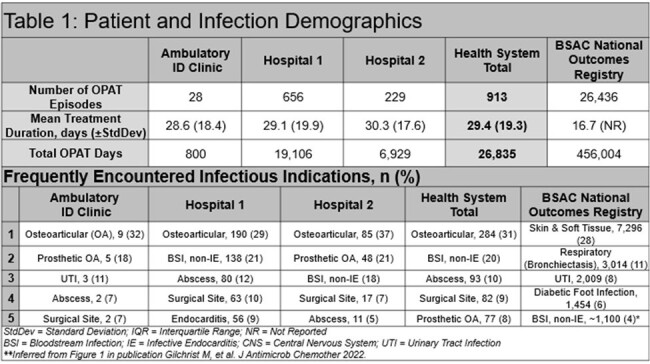

**Results:**

Overall, 913 OPAT episodes were identified. Infections observed at our institution were more invasive and required longer treatment durations (Table 1). Outcomes were known in 798 (87.4%) patients, with improvement/cure reported in 689 (86.3%) in comparison to 92.4% (number not reported) in the NORS registry (Table 2). Institutional performance relative to VAD-related events was within the ranges observed by both NORS (1.4 per 1000 OPAT days vs. 1.4 per 1000 OPAT days [reported range: 0.11 – 10.4]) and meta-analysis (Table 3). These rates remained comparable when further classified as either infection-related or CRT. In our OPAT cohort, rates of catheter removal events and medication errors were rare, occurring in 0.5 and 0.6 events per 1000 OPAT days, respectively. These were novel OPAT metrics not reported in the comparator registry.

**Figure:**
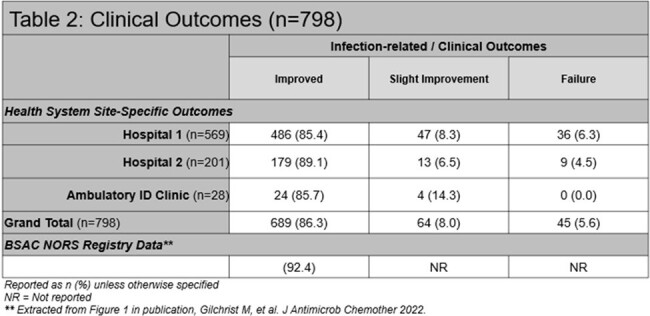

**Conclusion:**

Compared to the NORS national registry, infections managed with OPAT at our institution were more deep-seated and required longer durations of therapy. However, our reported clinical outcomes at the end of therapy remained comparable and were within reported ranges.

**Figure:**
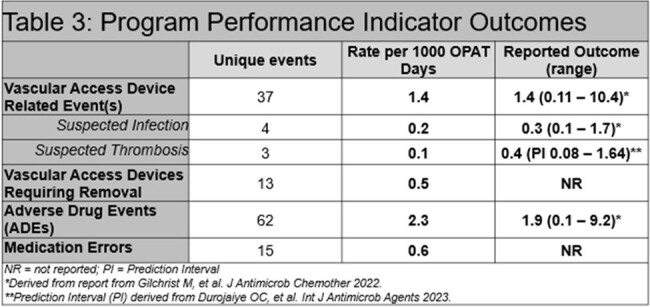

**Disclosures:**

Alisha Clemons, APRN, Gilead Sciences: Honoraria

